# Caring for the caregivers: Evaluation of the effect of an eight-week pilot mindful self-compassion (MSC) training program on nurses’ compassion fatigue and resilience

**DOI:** 10.1371/journal.pone.0207261

**Published:** 2018-11-21

**Authors:** Martin C. Delaney

**Affiliations:** 1 School of Education, University of Aberdeen, Aberdeen, Scotland, United Kingdom; 2 Blooming Mental Wellbeing, Clarinbridge, Galway, Ireland; University of Birmingham, UNITED KINGDOM

## Abstract

**Background:**

Nurses vicariously exposed to the suffering of those in their care are at risk of compassion fatigue. Emerging research suggests that self-compassion interventions may provide protective factors and enhance resilience. This pilot study examined the effect of an eight-week Mindful Self-Compassion (MSC) training intervention on nurses’ compassion fatigue and resilience and participants’ lived experience of the effect of the training.

**Methods:**

This observational mixed research pilot study adopted an evaluation design framework. It comprised of a single group and evaluated the effects of a pilot MSC intervention by analyzing the pre- and post-change scores in self-compassion, mindfulness, secondary trauma, burnout, compassion satisfaction, and resilience. The sample of the nurses’ (N = 13) written responses to the question, *“How did you experience the effect of this pilot MSC training*?*”* were also analyzed.

**Results:**

The Pre- to Post- scores of secondary trauma and burnout declined significantly and were negatively associated with self-compassion (r = -.62, p = .02) (r = -.55, p = .05) and mindfulness (r = -.54, p = .05). (r = -.60, p = .03), respectively. Resilience and compassion satisfaction scores increased. All variables demonstrated a large effect size: Mean (M) Cohen’s d = 1.23. The qualitative emergent themes corroborated the quantitative findings and expanded the understanding about how MSC *on the job* practices enhanced nurses’ coping.

**Conclusion:**

This is the first study to examine the effect of a pilot (MSC) training program on nurses’ compassion fatigue and resilience in this new area of research. It provides some preliminary empirical evidence in support of the theorized benefits of self-compassion training for nurses. However, further research, such as a Randomized Control Trial (RCT) with a larger sample size and a longitudinal study, is required to see if the benefits of self-compassion training are sustainable.

## Introduction

While empathy and compassion are often seen as two essential qualities in caregiving, continuous exposure to the suffering of others on a daily basis carries a risk of compassion fatigue in nurses [1]. This can impact a nurse’s professional ability, personal life, and potentially lead to an increase in staff shortages [2–3]. Multiple studies have identified that 40–60% of healthcare professionals are challenged by burnout at some stage of their career [3–4]. Compassion fatigue (CF) is defined as a state of exhaustion and dysfunction as a consequence of prolonged exposure to suffering and stress [5]. This research pilot study uses a model that conceptualizes CF as comprising of two main negative aspects secondary traumatic stress and burnout along with the positive aspect of compassion satisfaction which is defined as the fulfillment one gets from being able to do one’s work well. [5–6]. There is some consensus that burnout is a response to prolonged exposure to demanding interpersonal circumstances and is characterized by emotional exhaustion, depersonalization, and reduced personal accomplishment [6–7]. In a healthcare setting, burnout may be experienced by both back office staff and caregivers. Secondary traumatic stress, however, is a consequence of nurses’ and other caregivers’ daily exposure to their clients’ suffering. It comes about as a result of a countertransference of suffering from the clients to nurses as a result of an unconscious attunement to and absorption of the clients’ stresses and trauma. Moreover according to Bride secondary traumatic stress specifically is a phenomenon whereby nurses or other caregivers may become traumatized not by directly experiencing a traumatic event but by indirect exposure to the traumatic events and suffering being experienced by those in their care. .[6, 8–9,10].

Emerging research suggests that compassion skills training could serve as an antidote to nurses’ secondary trauma and burnout [4, 8]. A number of studies have examined the role of mindfulness-based interventions (MBis) [2,11] and loving kindness meditation interventions (LKMi) for caregivers [12]. Additionally, a meta-analysis of the association between self-compassion and psychopathology identified associations between higher self-compassion and lower psychopathology [13]. However, a review of the relevant literature till date (till November 2017) found no study that evaluated the effect of a self-compassion intervention (SCi) on nurses’ CF and resilience. The majority of the studies to date were correlational studies, cross-sectional surveys, or reviews of published literature [3–4, 14]. Therefore, this pilot study was the first step in gaining some *preliminary empirical evidence* on the theorized benefits of self-compassion skills training to nurses in a real-world setting.

Kristin Neff and Christopher Germer developed the Mindful Self-Compassion (MSC) intervention to provide participants with a variety of tools to enhance self-compassion and integrate it into their daily lives. They proposed that emerging research indicates a role for explicitly teaching self-compassion skills as a means of enhancing positive psychological health [15]. In addition, they suggested that greater self-compassion has been found to predict lower levels of anxiety and depression [15]. MSC as a group strengths based positive psychological approach could have potential advantages over the more usual method of dealing with nurses’ challenges in a one to one clinical setting. For example it is preventative rather than reactive and as a group intervention it should prove more cost effective than one to one therapy. Neff and Germer evaluated the effectiveness of their MSC intervention in a pilot study and a randomized controlled trial (RCT) and found that the MSC intervention was successful in enhancing participants’ psychological functioning and that participants also experienced greater life satisfaction and less anxiety and depression. [16]

### Study purpose

The pilot study sought to gain some *preliminary empirical evidence* to extend the initial research literature that theorized the benefits of self-compassion skills training to nurses. It chose to do this by assessing the effect of a pilot eight–week SCi in relation to nurses’ compassion fatigue and resilience. As there are no studies to date that examine the effects of a SCi on nurses’ compassion fatigue and resilience, any *preliminary empirical evidence* gathered may identify possible effects and associations that may be worth following up in subsequent larger studies. As a pilot study, this study is not a hypothesis testing study but a first step in exploring a novel intervention in the real-world setting of a busy university hospital with the goal of providing a *preliminary assessment* of the benefits of self-compassion skills training to nurses [17–18, 19, 20].

### Definitions and key concepts

#### Compassion and self-compassion

Compassion can be defined as a basic kindness with a deep awareness of the suffering of oneself and other living things, coupled with the wish and effort to relieve it [21]. It is an innate ability, a mental state capable of being enhanced through training [22]. Moreover, self–compassion gives one the ability to hold one’s feelings of suffering with a sense of warmth, connection, and concern, rather than reacting with self-criticism and self-judgment. It is a response that reverses the more usual reactions of avoidance and suppression of suffering [23]. Neff [24] conceptualizes self-compassion by contrasting its three aspects with three opposing mental states, as follows:

1. 1 *Self-kindness*: The ability to treat oneself with care and understanding rather than being harsh, judgmental, and self-critical.
2. *A sense of common humanity*: Recognizing that imperfection is a shared aspect of human experience rather than feeling isolated by one’s failures.
3. *Mindfulness*: Holding one’s painful thoughts and feelings in a balanced perspective rather than avoiding them or exaggerating the dramatic storyline of one’s suffering.

#### Mindfulness and compassion

According to Nairn [25], two aspects of mindfulness are a knowledge of what is happening in the present moment coupled with an attitude of non-preference to whatever arises in our field of awareness. However, mindfulness and compassion are seen as separate processes that need to be explicitly cultivated in their own ways though they have overlapping but quite different effects on the brain [22]. Both mindfulness and compassion involve moving toward discomfort or pain with an accepting rather than a conditioned reactive stance. However, the differences between the two are further clarified by Neff and Dahm [26] who stated that while mindfulness involves a balanced awareness of negative thoughts and feelings with equanimity, compassion has a broader scope that includes the elements of kindness and common humanity combined with the *actions* of actively soothing and comforting oneself or others. Like mindfulness, compassion training has a long tradition in Buddhist practice. Neale has compared the process of cultivating compassion through training to Wolpe’s theory of reciprocal inhibition [27]. This theory proposes that repeated practice establishes a desired attitude or behavior in direct antithesis to the stress response of flight, fight, or freeze when confronted with anxiety provoking stimuli. Reciprocal inhibition, according to Neale [27], is called *pratipakha bhavanam* in Sanskrit, meaning the cultivation of an opposing thought. From this perspective, training nurses to enhance their attitudes of self-compassion can be seen as an antidote to self-critical attitudes that can lead to the subordination of their own needs and an over-identification with patient outcomes.

Consequently, the study expected that nurses who attended a pilot eight-week MSCi would show enhanced internal resources of self-compassion and mindfulness and that these enhanced internal resources would be associated with a reduction in both negative aspects of compassion fatigue secondary traumatic stress and burnout, while also being associated with increased resilience and compassion satisfaction. Moreover, as a new area of investigation, the study examined nurses’ lived experience of participating in this pilot MSCi. The study was carried out in the Health Service Executive (HSE) of the Irish National Health Service at a university hospital in the west of Ireland.

## Method

### Study design

This observational pilot study adopted an evaluation research approach following STROBE guidelines [28]. The study had a single group evaluation design that used mixed research methods. A pilot study, according to Thabane et.al., has the possibility of finding *preliminary empirical evidence* that could inform decisions on the need for larger scale studies [17]. As a new area of investigation, the study combined a pragmatic stance with mixed research methods [29–30,31]. This allowed for a corroboration of results between the two data sets, gave a richer understanding of participants’ experience of the intervention, allowed for elaboration of the results beyond what could be known with the use of self report instruments alone, and enhanced the interpretation of the findings [32]. The mixed research framework was a partially mixed, concurrent design with a dominant quantitative phase [32]. The quantitative phase consisted of the calculation of descriptive and inferential statistics. The qualitative phase used a phenomenological approach based on Interpretative Phenomenological Analysis (IPA) [33–34]. The mixed methods phase, including data transformation and integration of the two data sets, followed the guidance of Leech and Onwuegbuzie whereby the qualitative data was quantitated and combined with quantitative data to form a coherent whole data set [32]. This design choice provided the best opportunity to answer the research questions in the context in which the study was taking place.

### Participants

Participants were all female nurses (n = 18). However, five participants did not complete the full eight-weeks MSC training, so the final sample size was n = 13. The sample size was just in excess of the sample size of 12 that is proposed for pilot studies by Julious, and by Belle, and also by the Royal College of Surgeons Ireland (RCSI) Guide for pilot studies [35–36, 37].

### Procedure

Prior to the commencement of the study, the study topic was reviewed and approved by the Irish Health Service Executive (HSE) regional ethics committee (REC) and the University of Aberdeen, where it was submitted as part of the completion of the MSc degree in mindfulness, compassion, and insight studies.

In order to have a varied sample a poster advertisement was broadcast electronically to each area of the hospital. After an information evening, participants were self-selected and completed informed consent forms.

The sample was representative with participants coming from across a range of the hospital’s disciplines including Cancer Care, Cardiology, Maternity, Midwifery, Intensive Care, and Urology. The sample of participants was also representative in relation to ages from 30< to >60 years and, likewise, experience pertaining to the number of years working in nursing from 10< to >31 years. The mean age of the sample was M = 44 years, and participants’ mean years of having worked as nurses was M = 25. Particularly relevant to this study was that 70% of the nurses in the sample had worked as nurses between 21–40 years, and, therefore, would have had exposure to secondary traumatic stress for a longer period of time than, for example, students or trainee nurses. None of the participants had any previous meditation experience.

Subsequently, participants attended a pilot eight-week MSCi that took place weekly during normal working hours and during times suitable for both day and night duty staff. All participants completed pre- and post-intervention measures on mindfulness, self-compassion, resilience, and compassion fatigue. Additionally exploratory qualitative data was gathered on week eight of the MSCi in a twin approach that combined a brief 40-minute focus group discussion and participants written responses to the question *“How did you experience the effects of this Pilot (MSC) training*?*”* [38–39].

### The intervention

This generic eight-week training teaches core principles and practices that enable participants to respond to difficult moments in their lives with kindness, care, and understanding. Participants attended a two-and-a-half hour training session each week and also participated in a half-day retreat. The focus of this MSC program was on helping participants develop self-compassion, with a secondary emphasis on mindfulness. In addition to weekly training sessions, participants were encouraged to practice on a daily basis and received four practice CDs of formal practices and informal practices that they could use *on the job*. The core practices of MSC include Mindfulness Meditation (MM), Loving Kindness Meditation (LKM), and Compassion Meditation (CM). A meditation session took place on each week of the course. The intervention teacher was a trained MSC teacher with the Center for Mindful Self-compassion (CMSC) and a fully accredited therapist/mental health professional in private practice, having previously worked in the Irish Health Service Executive (HSE) Employee Assistance Program (EAP). The choice of an eight–week MSC program is based on the fact that the majority of the evidence supporting the efficacy of mindfulness training is predominately based on the investigation of eight-week interventions [40]. In addition the eight-week MSC program is a manualized intervention, which will allow for repeatability and better comparisons with future research [41].

## Measures

Pre- and post-intervention quantitative data was gathered using the following standardized self-report instruments: The Neff Self–Compassion Scale 26 item, the Frieburg Short Mindfulness Scale, ProQOL Professional Quality of Life Scale, and Conor-Davidson Resilience Scale 25 Item.[42–43, 44,5].

**The Neff 26-item Self-compassion scale** was developed to measure both the negative and positive aspects of the three main components of self-compassion: self-kindness versus self-judgment, common humanity versus isolation, and mindfulness versus over-identification. This is based on Neff’s conceptualization of self-compassion, which was used in this study [24]. The internal consistency of the Neff 26 Item Self-compassion scale was Cronbach’s α = .97 [42].

**The Freiburg Mindfulness inventory,** a 14-item short version, was developed to measure mindfulness in people with no background in mindfulness or meditation and, for that reason, was appropriate for inclusion in this study. The internal consistency of the Freiburg Mindfulness Inventory was Cronbach’s α = .79 [43].

**ProQOL Version 5 Professional Quality of Life Scale: Compassion Satisfaction and Fatigue Version** [5]: ProQOL measures both the negative and positive effects of helping people who experience suffering and trauma. The ProQOL has sub-scales for compassion satisfaction, burnout, and the secondary traumatic stress associated with caregiving. The measure has been in use since 1995. Several revisions have been brought about in this measure with ProQOL 5 being the current version. The scales are:

*Compassion Satisfaction*: This is about the fulfillment one derives from being able to do one’s work well. For example, one may feel like it is a pleasure to help others through one’s work. The internal consistency of the ProQOL Compassion Satisfaction scale was Cronbach’s α = . 87.

*Burnout*: From the research perspective, this is one of the elements of compassion fatigue (CF). It is associated with feelings of hopelessness and difficulties in dealing with work or in effectively doing one’s job. The internal consistency of the ProQOL Burnout Scale was Cronbach’s α = .72.

*Secondary Traumatic Stress*: The second component of compassion fatigue is work-related secondary exposure to extremely or traumatically stressful events. The internal consistency of the Pro QOL Secondary Traumatic stress scale was Cronbach’s α = .80.The three scales cannot be combined to give an overall compassion fatigue score. However, it is designed to measure both the positive and negative effects of being a caregiver, which made these scales very relevant to this study.

**Connor-Davidson Resilience Scale 25 item (CD-RISC 25)** is designed to measure a person’s ability to cope with stress and adversity. It is based on the definition of resilience as the ability to adapt well, overcome adversity, and even thrive in the face of adversity. The fact that this scale was developed to assess the positive effects of treatment for stress made it relevant in assessing the effects of this study’s intervention on participants’ resilience. The internal consistency of the CD-RISC 25 was Cronbach’s α = .89 [44].

A summary of the tools used along with their reliability and validity is shown in Table 1 below.

**Table 1:** Summarizing the measures used with their reliability and validity.

Scale	Cronbach Alpha	Validity
**Neff. 26 item Self-Compassion Scale**	a = .97	Factor Analysis results support a six factor model. Positive correlations with instruments measuring similar constructs
6 Factor Sub-scales		
Self-kindness	a = .79	
Self-judgement	a = .74	
Common humanity	a = .72	
Isolation	a = .69	
Mindfulness	a = .85	
Over-Identification	a = .72	
**Frieburg Short Mindfulness Scale**	a = .79	Positive Correlations between this and other instruments measuring mindfulness across a multitude of samples
**Proqul Professional Quality of Life Scale**		
		There is good construct validity with over 200 published papers.Nearly half of the 100 published research papers on compassion fatigue and secondary traumatic stress have utilised this scale
Sub-scales		The sub-scales cannot be combined. The three scales measures separate constructs
Compassion Satisfaction	a = .87	
Burnout	a = .72	
Secondary Traumatic Stress	a = .80	
**Connor—Davidson Resilience Scale 25 Item**	a = .89	Positive correlation with instruments measuring similar constructs. r = .82

The measures used in the study were all relevant to the studies’ conceptualizations and also relevant to the sample. For example the ProQol measure corresponded to the studies conceptualization of compassion fatigue, and the Frieburg Short Mindfulness Scale was appropriate for participants with no previous meditation experience. According to Simmons and Lehmann all social science instruments have a degree of error however as shown below all the measures chosen for this study had high levels of reliability and therefore less error. The reliability of the measures was characterized by good internal consistency that ranged from cronbach alpha a = .72 for burnout to cronbach alpha a = . 97 for self-compassion. Likewise the measures have demonstrated high validity with positive correlations with other instruments measuring similar constructs. [45]

Consequently all the measures used were relevant to the study and had high levels of reliability and validity.

### Quantitative data analysis

Descriptive Statistics were calculated including the means and standard deviation for all variables pre- and post-intervention and Cohen d to measure effect size. Inferential statistics were used to examine the relationships between independent and dependent variables. Data was plotted into scatter plots and the following inferential statistics were used: Pearson’s correlation represented by r = to measure the strength and direction of associations between independent variables, self-compassion and mindfulness and the dependent variables, secondary traumatic stress, burnout, compassion satisfaction, and resilience. Linear regression was used to calculate the extent to which the variance in the dependent variables was attributable to the independent variables, represented by R^2^. P values are inserted for the benefit of the reader and not in an attempt at hypothesis testing [46–47,48].

### Qualitative data analysis

A phenomenological approach based on interpretative phenomenological analysis (IPA) was used in order to gain an understanding of the group’s shared lived experience of participating in this pilot MSCi. IPA is concerned with the subjective reports of individuals rather than the formulation of objective accounts. There are dual aspects to IPA: the reflections of the participants and the interpretative analysis of the researcher [33–34]. So, in order to gain a deeper understanding of what participating in this pilot program meant for the group, a brief 40-minute focus group session was held on completion of the intervention. This consisted of a group discussion among the participants on the question, *“How did you experience the effects of this pilot (MSC) training*?*”* During the group discussion, the nurses shared reflections and perspectives on their experiences. The eight-week MSC program contained a number of guided reflection exercises to help participants gain a deeper insight into their own experience. Likewise, at the end of the focus group discussion, participants were invited to again reflect on the question, *“How did you experience the effects of this pilot (MSC) training*?*”* They then wrote down what they discovered, and the written responses gathered in this way was the source of the qualitative data. Subsequently, participants’ written reports of their lived experience were subject to a subjective and reflective interpretation by the researcher. Themes that emerged directly from the data were initially coded and categorized into broad themes and were recoded into more specific emergent themes after continuous reading. Magnitude Coding was used to indicate the frequency and importance of the emerging themes and to facilitate qualitative data mixing with the quantitative data [38–39].

### Data mixing

Percentile rank was calculated on the frequency of participants’ responses in relation to each of the qualitative emergent themes to denote the importance of each emergent theme, on the following basis: 3 = High, based on whether they were on or above 75 percentile, 2 = Medium, between 25–74 percentile, and 1 = Low, below 25 percentile. These quantitated qualitative results were then combined with the quantitative results, resulting in the formation of a combined data set. This data mixing facilitated the corroboration of the results from individual data sets. Furthermore, data mixing also facilitated further insights from the results than those that would have been gained with the use of pre-determined variables alone. It also enabled elaboration on the issues arising from combining data [29, 39].

## Results

### Quantitative data

A summary of the main quantitative findings pre–to- post the MSC intervention are shown in Table 2 below. It shows the trends for the independent variables self-compassion and mindfulness and the dependent variables secondary traumatic stress, burnout, compassion satisfaction, and resilience.

**Table 2:** Summary of the group’s pre- and post-intervention mean scores and effect size using cohen’s d.

Variables	Pre. Mean	S.D	Post.Mean	S.D	Effect Size Cohen d	95% Confidence Interval Cohen d
Self-compassion	2.87	0.67	3.57	0.38	1.28	[0.1–2.48]
Mindfulness	33.92	6.56	42.00	4.86	1.40	[0.19–2.61]
Secondary Traumatic Stress	27.23	4.10	23.84	4.21	0.82	[-1.9–0.32]
Burnout	29.07	4.34	23.07	3.35	1.55	[-2.79–0.31]
Compassion Satisfaction	37.92	3.45	41.00	3.94	0.83	[-0.30–1.97]
Resilience	67.61	8.79	80.30	8.08	1.50	[0.27–2.73]

The analysis of the quantitative data indicated that post intervention scores on the self-compassion, mindfulness, compassion satisfaction and resilience scales were increased. Moreover the groups’ reported scores on the scales representing the two negative aspects of compassion fatigue, secondary traumatic stress and burnout both showed decreases. Effect size Cohen d for all the variables was large between d = .82 [CI -1.9–0.32] to d = 1.5[CI 0.27–2.73] in accordance with Cohen table for the interpretation of effect size. Mean effect size was (M) d = 1.23. [46–47].

Overall participants’ reported scores showed an increase for self-compassion, mindfulness, compassion satisfaction and resilience. Whereas reported scores in both of the negative aspects of compassion fatigue secondary traumatic stress and burnout decreased.

The analysis shown in Table 3 below reveals the strength and direction of the relationship between self-compassion and secondary traumatic Stress, burnout, compassion satisfaction, and resilience.

**Table 3:** Correlation analysis using pearson correlation showing the strength and direction of the relationship between self-compassion and secondary traumatic stress, burnout, compassion satisfaction, and resilience.

Variables	Pearson Correlation r =	P-value =
Secondary Traumatic Stress	-0.62	0.02
Burnout	-0.55	0.05
Compassion Satisfaction	-0.19	0.52
Resilience	+0.27	0.37

*Note*. The guidance used for interpreting the Pearson’s correlation was: Small = ± .1 to .29, Medium ± .3 to .49, and large ± .5 to 1.0 [48]

There was a strong negative association between participants’ reported scores of self-compassion and both negative aspects of compassion fatigue, secondary traumatic stress r = -0.62 and burnout r = 0.55 respectively. Of particular interest is the fact there was just a small positive association between self-compassion and compassion satisfaction and resilience. However this was a pilot study with a small sample size. A study with a larger sample size may for example find that an association of r = 0.27 between self-compassion and resilience might become significant.

Self-compassion had a negative association with both negative aspects of compassion fatigue secondary traumatic stress and burnout. However the association of self-compassion with compassion satisfaction and resilience requires further investigation.

The strength and direction of the relationship between mindfulness and secondary traumatic Stress, burnout, compassion satisfaction, and resilience can be seen in Table 4 below.

**Table 4:** Correlation analysis using pearson correlation showing the strength and direction of the relationship between mindfulness and secondary traumatic stress, burnout, compassion satisfaction, and resilience.

Variables	Pearson Correlation r =	P-value =
Secondary Traumatic Stress	-0.54	0.05
Burnout	-0.60	0.03
Compassion- Satisfaction	-0.25	0.41
Resilience	+0.66	0.01

*Note*. The guidance used for interpreting the Pearson’s correlation was: Small = ±. .1 to .29, Medium ± .3 to .49, and Large ± .5 to 1.0 [48]

The results indicated a strong negative association between participants’ reported enhanced mindfulness and secondary traumatic stress and burnout, wherein with an increase in mindfulness, there was a decrease in secondary traumatic stress and burnout. These reported results also show a large positive association between mindfulness and resilience r = 0.66. However, once again, there was no statistically significant relationship between mindfulness and compassion satisfaction with a p value of .41

Mindfulness had a negative association with both secondary traumatic stress and burnout. While there was a large positive association between mindfulness and resilience once more further investigation is required on the association between mindfulness and compassion satisfaction.

A summary of simple linear regression showing how the dependent variables were associated with self-compassion is presented in Table 5 below.

**Table 5:** A Summary of simple linear regression showing how the dependent variables were associated with self-compassion.

Variables	Coefficient of Determination R^2^	β	F	p-level
Secondary Traumatic Stress	.39	-6.77	6.9	.02
Burnout	.30	-4,73	4.66	.05
Compassion Satisfaction	.04	-1.98	.43	.52
Resilience	.07	5.6	.87	0.37

The results indicated a negative trend a decrease in reported secondary traumatic stress and burnout as self-compassion increased. Reported enhanced self-compassion was a predictor of reduced secondary traumatic stress **β = -**6.77 and burnout **β = -**4.73 respectively. However, once more self-compassion had no statistically significant association with either compassion satisfaction or resilience.

Reported enhanced self-compassion was a predictor of reduced secondary traumatic stress and burnout.

However the association between self-compassion and compassion satisfaction and resilience requires further investigation.

A summary of simple linear regression describing how the dependent variables were associated with mindfulness is shown in Table 6 below.

**Table 6:** A Summary of linear regression showing how the dependent variables were associated with mindfulness.

Variable	Coefficient of Determination R^2^	β	F	p-level
Secondary Traumatic Stress	.30	-.47	4.62	.05
Burnout	.36	-.42	6.28	.03
Compassion Satisfaction	.06	-.2	.72	.41
Resilience	.44	1.09	8.47	0.01

These results indicated a negative trend whereby as the groups’ reported score in mindfulness increased, there was a decrease in reported group scores for secondary traumatic stress and burnout. Moreover enhanced mindfulness was a predictor of reduced secondary traumatic stress **β** = -.47 and also burnout **β = -.**42. Conversely the increased group reported score for mindfulness was predictor of a large increase in enhanced resilience **β** = 1.09. However once again there was no statistically significant association with compassion satisfaction, indicating again that this requires further investigation.

Reported enhanced mindfulness was a predictor of reduced secondary traumatic stress and burnout and enhanced resilience. However the association between mindfulness and compassion satisfaction requires further investigation.

### Qualitative data

**Fig 1:**
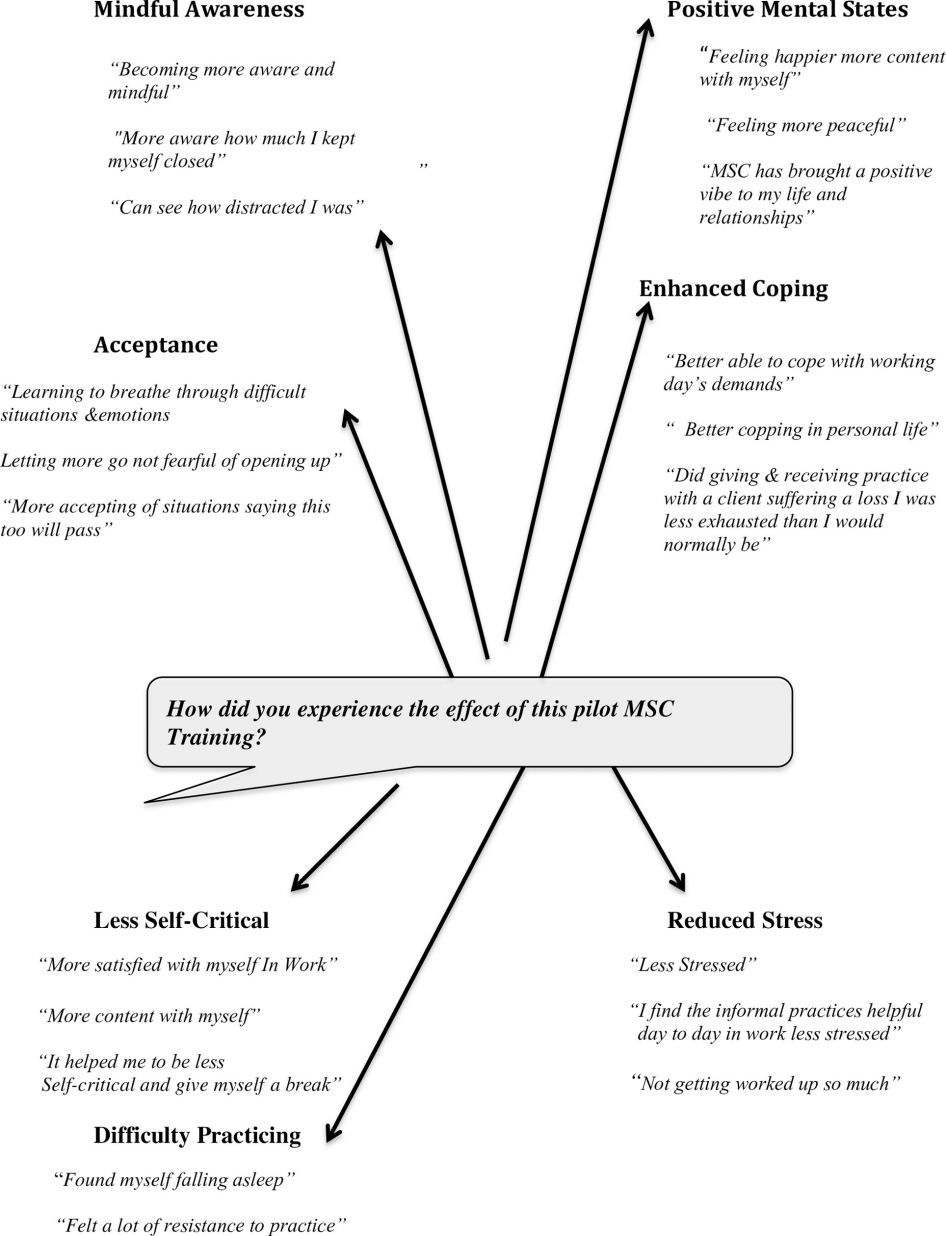
Dialogue map of the emergent themes and a sample of participants’ responses [49].

Analysis of the exploratory qualitative data and the ranking of the emergent themes according to their importance is shown in Table 7 and Fig 1 below.

**Table 7:** Showing the emergent themes ranked according to their importance.

Emergent Themes	Importance of Emergent Themes based on Participants’ Responses
Positive Mental States	3
Enhanced Coping	3
Acceptance	2
Mindful Awareness	2
Less Self-critical	2
Reduced Stress	1
Difficulty Practicing	1

*Note*. Percentile rank was calculated on the frequency of participants’ responses associated with each of the emergent themes to denote the importance of each emergent theme, on the following basis: 3 = High, based on whether it was on or above 75 percentile, 2 = Medium, between 25–75 percentile, and 1 = Low, below 25 percentile [39].

The three medium ranked emergent themes of *Acceptance*, *Mindful Awareness*, *and Less Self-Critical*, could be seen to represent aspects of participants’ enhanced skills of self-compassion and mindfulness. The two highest ranked emergent themes of *Positive Mental States*, *Enhanced Coping* along with one of the low ranked emergent theme *Reduced Stress may* be associated with these enhanced skills. Interestingly even though none of the nurse participants’ had any previous meditation experience the emergent theme *Difficulty Practicing* ranked as of low importance.

Emergent themes can be viewed as being associated with aspects of participants’ enhanced skills of self-compassion and mindfulness and also associated with participants’ improved positive affect and coping skills.

Fig 1 below shows the relationship between emergent themes and an example of participants’ responses to the focus group question, *“How did you experience the effect of this pilot MSC training*?*”*

### Data mixing

Qualitative and Quantitative Data was combined in Tables 8 and 9 below, allowing for a corroboration and elaboration of the results.

**Table 8:** The emergent themes associated with their nearest variable.

Emergent Theme	Independent Variable	Dependent variable
Mindful Awareness	Mindfulness	
Acceptance	1.Mindfulness & 2.Selfcompassion	
Less Self-critical	Self-compassion	
Enhanced coping		Resilience
Reduced stress		Secondary Traumatic Stress & Burnout
Positive Mental states	None	None
Difficulty Practicing	None	None

**Table 9:** Combined results of emergent themes and associated variables pre- and post-intervention scores and effect size.

Associated Emergent theme	Importance Of Theme	Variable	Pre.	Post.	Effect Size 95% Cohen’s d C.I.
Less Self-critical	2	Self-compassion	2.87	3.57	1.28 [0.1–2.48]
Mindful Awareness	2	Mindfulness	33.92	42	1.40 [0.19–2.61]
Acceptance	2	1. Self-compassion 2. Mindfulness	2.87 33.92	3.57 42	1.28 [0.1–2.48] 1.40 [0.19–2.61]
Reduced Stress	1	Secondary Traumatic Stress	27.23	23.84	.82 [-1.9–0.32]
Reduced Stress	1	Burnout	29.07	23.07	1.55 [-2.79–0.32]
None	0	Compassion Satisfaction	37.92	41	.83 [-0.33–1.97]
Enhanced Coping	3	Resilience	67.61	80.30	1.5 [0.27–2.73]
Positive Mental States	3	None			
Difficulty Practicing	1	None			

The outcome of combining the emergent themes with their nearest corresponding variable is shown in Table 8 below.

The majority of the emergent themes could be seen to be associated with a pre-determined variable.

The exception being *Difficulty Practicing* which was of low importance and also *Positive Mental States* which was of high importance. While *Positive Mental States* was not associated with a pre-determined variable this finding does concur with the findings of Neff et al., that training in self-compassion was associated with increased happiness optimism and positive affect.

The emergent themes were closely associated with the pre-determined variables. The main exception *Positive Mental States* concurred with the findings of Neff et al., in a study that examined self-compassion in relation to positive psychological functioning. **[50]**

The combined quantitative and qualitative results are shown in in Table 9 below.

The qualitative data results corroborated the quantitative finding. Generally the importance of emergent themes and effect size were related in that a theme’s ranking of importance was associated with the relevant effect size. For example *Enhanced Coping* was of high importance and was associated with Resilience the variable with one of the largest effect size Cohen d = 1.5 [C. I. 0.27–2.73]. However the other emergent theme of high importance *Positive Mental States* was not associated with a pre–determined variable. Nevertheless, the *Positive Mental States* reported by participants could be expected to have an impact on *Compassion Satisfaction*, the fulfillment one gets from being able to help others through one’s work. Once more this is an issue requiring further examination.

The combined data set demonstrated that the exploratory qualitative data corroborated the quantitative findings.

## Discussion

### Independent variables self-compassion and mindfulness

This study sought to find some preliminary evidence in a real-world setting of a busy university hospital in relation to the theorized benefits of self-compassion training to nurses [6–7]. This was approached by examining the effects of a pilot eight-week MSCi on nurses’ compassion fatigue and resilience. The results suggested that this pilot MSCi was associated with participants’ reported enhanced capacities of self-compassion and mindfulness. The group’s (.70) increase on the self-compassion scale (SCS) and a 8.08 increase on the mindfulness scale with a large effect size (d = 1.28 95% C.I [.0.1–2.48] and 1.40 95% CI [.0.19–2.61]), respectively, concurs with Neff and Germer’s [16] randomized controlled trial (RCT) of MSC where they also found a large effect size in both self-compassion and mindfulness Cohen’s d = 1.60 and .60, respectively. In comparison, they quoted three studies on Mindfulness Based Stress Reduction (MBSR) [51–52, 53] that yielded an average effect size of d = . 54 increase on the SCS. An interesting difference between this study and the Neff and Germer’s RCT is the reversal of increased scores and effect size for self-compassion and mindfulness. A possible explanation is the very different samples. Neff and Germer stated that in their sample, 81% of participants had previous meditation experience, while in this study, none of the nurses had any previous meditation experience. [16]

### Associated qualitative emergent themes

Additionally, the quantitative results were supported by the qualitative analysis of participants’ responses to the focus group question, “*How did you experience the effects of this Pilot MSC training*?” Emergent themes closely associated with self-compassion and mindful awareness both ranked as having medium importance. Likewise acceptance also ranked as having medium importance. Acceptance is more often seen as an aspect of mindfulness [25, 54]. But Baer et al. [55] expanded the view of acceptance to incorporate both non-reactivity and non-judgment. Two of the six components of self-compassion, according to Neff [24], are the positive aspects of self-kindness rather than a negative reactivity of being harsh, judgmental, and self-critical. Therefore, from this perspective, acceptance can be seen as a meeting point between mindfulness and self-compassion. It indeed features in all of the core practices taught in MSC. Accordingly, the results from both quantitative measures and the qualitative analysis suggested that this pilot MSCi was associated with an enhancement of the nurses’ capacities of self-compassion and mindfulness.

### Dependent variables: Secondary traumatic stress

Secondary traumatic stress and burnout are seen as two critical aspects of compassion fatigue that are related to caregivers’ psychological problems [5, 9]. Secondary traumatic stress is defined as the stress resulting from helping or wanting to help a traumatized or suffering person [6]. There was a significant reduction in nurses’ baseline score for secondary traumatic stress of 3.39 from pre (M = 27.23) to a post score of (M = 23.84) representing a large effect size of d = .82 95% CI [-1.9–0.32]. This can be compared with Potter et al. [56], who evaluated a five-week compassion fatigue resiliency program for oncology nurses (n = 13) and reported a 2.15 reduction in secondary traumatic stress. While no other comparable empirical studies were found till date (November 2017), this concurred with the theorized benefits that proposed that self-compassion may be a beneficial protective factor in caregivers’ compassion fatigue [3,8]. The findings supplement these theories with some preliminary empirical evidence. As both self-compassion and mindfulness scores increased, secondary traumatic stress decreased, showing a strong negative association r = -.62 p <0.02 and r = -.54 p<0.05, respectively. Moreover, simple linear regression showed that the variance in self-compassion accounted for 39% (R^2^ = .39) of the variance in nurses’ secondary traumatic stress, while the variance in mindfulness accounted for almost 30% (R^2^ = .30) of the variance in secondary traumatic stress. This demonstrates that both self-compassion and mindfulness contributed to a significant reduction in nurses’ secondary traumatic stress. Additionally, participants’ increased capacities of both self-compassion and mindfulness were predictors of reduced traumatic stress, as represented by **β** = -6.77 and **β** = -.47, respectively. Participants’ responses in the qualitative data supported the quantitative data with two emergent themes: *Enhanced Coping and Reduced Stress* ranked as high importance and low importance, respectively.

### Burnout

As stated above, burnout is defined as a response to prolonged exposure to demanding interpersonal situations and is characterized by emotional exhaustion, depersonalization, and reduced personal accomplishment [7]. The study found a significant reduction of 6 in participants’ baseline burnout score from pre M = 29.07 to post M = 23.07, again representing a large effect size, Cohen’s d = 1.55, 95% CI. [-2.79–0.32]. This is compared with Potter et al. [56], who reported a reduction in oncology nurses’ burnout of .85 (pre, M = 23.46 to post M = 22.61). Once again, no comparable studies were found (November 2017) for further comparisons. However, this study identified negative associations between self-compassion and burnout, r = -0.55, and mindfulness and burnout, r = -0.60. Similarly, both enhanced self-compassion and mindfulness were predictors of reduced burnout, as represented by **β** = -4.73 and **β** = 0.41, respectively. Likewise, simple linear regression identified that self-compassion accounted for 30% of the variance in burnout (R^2^ = . .30) and mindfulness accounted for 36% of the variance in burnout (R^2^ = .36). It is noteworthy that the combined reduction in both aspects of compassion fatigue had an average effect size of Cohen’s d = 1.18, almost double the effect size of d = . .65 reported for various forms of therapy in a meta-analysis study, by Cuijpers et.al. [57]. Qualitative data once more supported the quantitative results. Two emergent themes, *Enhanced Coping* and *Less Stressed* were indicators of participants’ reduced emotional exhaustion and were ranked as high and low importance, respectively.

### Compassion satisfaction

Compassion satisfaction is defined as the fulfillment one derives from being able to do one’s work well and should be included in any assessment of nurses’ professional quality of life, according to

Stamm [5]. However, no statistically significant association was found between self-compassion or mindfulness and compassion satisfaction. But participants’ score did show a positive change from pre mean score (M = 37.92) to post mean score (M = 41), indicating an increase in compassion satisfaction of 3.95 with a large effect size Cohen’s d = .83 95% CI. [-0.33–1.97]. Likewise, Potter et al. found no statistically significant association between a compassion fatigue intervention and oncology nurses’ compassion satisfaction by [56]. One possible explanation of nurses’ increased compassion satisfaction in this study is the *Hawthorne Effect*, a psychological phenomenon that produces an improvement in human behavior or performance, as a result of increased attention from superiors, clients, or colleagues [58]. However, while no association was found between the pre-defined variables, qualitative results may give further insight into nurses’ increased compassion satisfaction. For example, it may be associated with the two highest ranked emergent themes *Positive Mental States and Enhanced coping*. This is obviously an issue that requires further examination in the future.

### Resilience

Resilience results showed a large effect size of Cohen’s d = 1.5 95% CI [.0.27–2.73]. The nurses’ pre-intervention resilience score of M = 67.61 was well below the mean (M = 80.4), found in the original report of a US general population sample by Connor and Davidson [44]. However, participants’ post mean score (M = 80.30) brought them into line with the general population norms. The results also demonstrated a strong positive association between the mindfulness aspect of this pilot MSCi and resilience (r = .66). In comparison, a survey of n = 45, Medicine Pediatric Residents [4] found mindfulness to have a moderate association with resilience (r = .38). Simple regression found that participants’ increased mindfulness explained a significant proportion of the variance in resilience (R^2^ = .44) and was also a predictor of increased resilience (**β** = 1.09 p = .01). Unexpectedly, however, no statistically significant association was found between participants’ self-compassion scores and resilience. This may be due to the small sample size (N = 13) and an association of r = . 27, may become significant in a larger sample. Significantly, a study by pediatric residents [4] showed a moderate positive association between self-compassion and resilience (n = 45, r = .37). However, it used a different resilience measure [59]. This is an issue that bears further investigation.

In relation to resilience, the qualitative results supported these quantitative findings. Resilience, according to Connor and Davidson, embodies personal qualities that enable one to thrive in the face of adversity and may be viewed as a stress coping ability [44]. The emergent theme *Enhanced Coping* was ranked as having high importance. Furthermore, additional details regarding this result emerged on an examination of the participants’ responses. Noteworthy is the fact that 80% of the nurses’ responses associated with this emergent theme related to their use of informal MSC practices *on the job*. For example, *“Informal practice in work allows me to step back from a situation”*, *“Practice is helpful in work”*, *“Practice in work made me less exhausted than I would normally be*.*”* This evidence suggested that these practices provided protective factors that could be used during the caregiving process rather than just after the event’s self-care strategies. However, further research is required to fully understand the mechanisms involved in these preliminary positive outcomes.

### Emergent theme: Positive mental states

Overall, the qualitative data in addition to a corroboration of the quantitative results gave a deeper understanding of participants’ lived experience of the effects of this pilot MSCi than what would have been achieved with the use of standard self-report instruments alone. Noteworthy is the fact that one of the two emergent themes ranked as having high importance was *Positive Mental States*. While not linked to any particular pre-defined variable, this qualitative result is in line with Cayoun who saw that a further benefit of training in positive mental attitudes, such as compassion, is that it creates the circumstance for the co-emergence of *Positive Mental States*. Co-emergence, according to Cayoun simply means two or more things emerging at the same time [60]. Therefore, training in compassion can be seen as creating the conditions that *may* lead to the co-emergence of other *Positive Mental States*. Furthermore, according to Hanson a repetition of the experience of *Positive Mental States* can lead to the cultivation of positive mental traits [61].

An association between LKM and CM and Positive Mental States has also been found in a number of previous studies. Self-compassion predicted significant variance in positive psychological health and strengths and was associated with happiness, optimism, and a positive effect, in a study by Neff et al. [50].Likewise Frederickson proposes that, positive emotions can broaden an individual’s momentary thought-action repertoire toward specific actions, in contrast to negative emotions that have a narrow focus, usually on survival involving flight, fight, or freeze reactions. By broadening the momentary thought-action repertoire, positive emotions loosened the hold of negative emotions [62–63].

### Study limitations and future directions

This pilot study included a small sample size (N = 13) and lacked a control group. These factors limited the extent to which the findings can be generalized, and although all variables had a large effect size, the study did not have a longitudinal phase to see if gains were maintained. However, to gain some preliminary insight into the practical significance of a SCi for nurses, this pilot study adopted a pragmatic approach. By evaluating a pilot MSCi using mixed research, the study was able to corroborate the results of both quantitative and qualitative data. Furthermore, the use of qualitative data enabled a greater insight into the benefits of *on the job* informal MSC practices to nurses.

Consequently, this pilot study, for the first time, obtained some *preliminary empirical evidence* in support of the theories that suggest that a SCi may benefit nurses in relation to compassion, fatigue, and resilience. Likewise, this pilot study, which involved a preliminary assessment of the benefits of a SCi to nurses, suggests that it is a promising area for future study. Further work required include an RCT with a larger sample size of nurses and an RCT with a larger sample size of other professional caregivers, such as social workers and clinicians. A longitudinal study would be beneficial to see if gains are maintained. In addition, it would be interesting to further examine the unexpected findings of this study, the association between self-compassion and resilience, and the role of self-compassion training in promoting caregivers’ *Positive Mental States* and their association with perceived benefits.

## Conclusions

This is the first study to examine the suggested theoretical benefits of a self-compassion intervention on nurses’ compassion fatigue and resilience. This observational pilot study is limited, in generalization, due to the small sample size and a lack of a control group. However, by evaluating the effects of a pilot Mindful Self-compassion Intervention for nurses using a mixed research approach, it has, for the first time, provided some preliminary empirical evidence of the practical significance of a SCi in providing nurses with *on the job* protective factors against compassion fatigue and for significantly enhancing their resilience. Moreover, the qualitative results suggested that training nurses in positive attitudes of love, kindness, and compassion may be associated with the co-emergent *Positive Mental States* that were reported.

## Supporting information

S1 Table Quantitative data scores. (DOCX)

S2 Table Quantitative data scores. (DOCX)

S3 Table Quantitative data scores. (DOCX)

S4 Table Qualitative data coding. (DOCX)

S5 Table Qualitative data coding. (DOCX)
