# Supplementary material for: Caring for the caregivers: Evaluation of the effect of an eight-week pilot mindful self-compassion (MSC) training program on nurses’ compassion fatigue and resilience
Source: PLoS One. 2018 Nov 21;13(11):e0207261. doi: 10.1371/journal.pone.0207261 (PMC6248952; doi:10.1371/journal.pone.0207261)
Supplement: S1 Table — (DOCX) [file pone.0207261.s001.docx]

| **Quantitative Data Scores** |  |  |  |  |  |  |  |  |  |  |  |  |  |  |  |  |  |  |  |  |  |  |  |  |  |  |  |  |  |  |  |  |  |  |
| --- | --- | --- | --- | --- | --- | --- | --- | --- | --- | --- | --- | --- | --- | --- | --- | --- | --- | --- | --- | --- | --- | --- | --- | --- | --- | --- | --- | --- | --- | --- | --- | --- | --- | --- |
| **Participant** | **Frieburg Scale Pre:** | **Q1** | **Q2** | **Q3** | **Q4** | **Q5** | **Q6** | **Q7** | **Q8** | **Q9** | **Q10** | **Q11** | **Q12** | **Q13** | **Q14** | **Participant** | |  |  |  |  |  |  |  |  |  |  |  |  |  |  |  |  |  |
| **No** |  |  |  |  |  |  |  |  |  |  |  |  |  | **REVERSE** | | **total** |  |  |  |  |  |  |  |  |  |  |  |  |  |  |  |  |  |  |
| 1 |  | 4 | 4 | 4 | 4 | 3 | 2 | 4 | 4 | 1 | 1 | 4 | 1 | 3 | 3 | 42 |  |  |  |  |  |  |  |  |  |  |  |  |  |  |  |  |  |  |
| 2 |  | 4 | 2 | 3 | 3 | 2 | 1 | 2 | 2 | 1 | 2 | 2 | 2 | 3 | 2 | 31 |  |  |  |  |  |  |  |  |  |  |  |  |  |  |  |  |  |  |
| 4 |  | 2 | 2 | 3 | 2 | 3 | 1 | 2 | 2 | 2 | 2 | 2 | 1 | 2 | 3 | 29 |  |  |  |  |  |  |  |  |  |  |  |  |  |  |  |  |  |  |
| 5 |  | 4 | 1 | 2 | 2 | 3 | 2 | 2 | 4 | 3 | 2 | 2 | 2 | 1 | 1 | 31 |  |  |  |  |  |  |  |  |  |  |  |  |  |  |  |  |  |  |
| 6 |  | 2 | 3 | 2 | 2 | 3 | 1 | 1 | 3 | 2 | 3 | 2 | 2 | 3 | 1 | 30 |  |  |  |  |  |  |  |  |  |  |  |  |  |  |  |  |  |  |
| 8 |  | 3 | 2 | 3 | 3 | 3 | 1 | 3 | 3 | 3 | 3 | 3 | 2 | 3 | 1 | 36 |  |  |  |  |  |  |  |  |  |  |  |  |  |  |  |  |  |  |
| 9 |  | 3 | 2 | 3 | 2 | 3 | 2 | 2 | 3 | 1 | 2 | 2 | 1 | 2 | 1 | 29 |  |  |  |  |  |  |  |  |  |  |  |  |  |  |  |  |  |  |
| 10 |  | 3 | 3 | 4 | 3 | 4 | 3 | 4 | 4 | 3 | 4 | 4 | 4 | 2 | 2 | 47 |  |  |  |  |  |  |  |  |  |  |  |  |  |  |  |  |  |  |
| 12 |  | 4 | 2 | 3 | 2 | 4 | 2 | 3 | 3 | 1 | 1 | 2 | 3 | 2 | 3 | 35 |  |  |  |  |  |  |  |  |  |  |  |  |  |  |  |  |  |  |
| 13 |  | 3 | 2 | 2 | 3 | 3 | 2 | 3 | 3 | 3 | 3 | 2 | 2 | 2 | 2 | 35 |  |  |  |  |  |  |  |  |  |  |  |  |  |  |  |  |  |  |
| 14 |  | 4 | 3 | 4 | 3 | 3 | 2 | 3 | 3 | 3 | 3 | 3 | 2 | 3 | 2 | 41 |  |  |  |  |  |  |  |  |  |  |  |  |  |  |  |  |  |  |
| 15 |  | 3 | 1 | 1 | 1 | 4 | 1 | 3 | 1 | 1 | 1 | 1 | 1 | 1 | 2 | 22 |  |  |  |  |  |  |  |  |  |  |  |  |  |  |  |  |  |  |
| 16 |  | 3 | 3 | 2 | 2 | 3 | 3 | 3 | 2 | 2 | 2 | 2 | 1 | 3 | 2 | 33 |  |  |  |  |  |  |  |  |  |  |  |  |  |  |  |  |  |  |
|  | **Group Mean** |  |  |  |  |  |  |  |  |  |  |  |  |  |  | **33.92** |  |  |  |  |  |  |  |  |  |  |  |  |  |  |  |  |  |  |
|  |  |  |  |  |  |  |  |  |  |  |  |  |  |  |  |  |  |  |  |  |  |  |  |  |  |  |  |  |  |  |  |  |  |  |
| 17 |  |  |  |  |  |  |  |  |  |  |  |  |  |  |  |  |  |  |  |  |  |  |  |  |  |  |  |  |  |  |  |  |  |  |
| 18 |  |  |  |  |  |  |  |  |  |  |  |  |  |  |  |  |  |  |  |  |  |  |  |  |  |  |  |  |  |  |  |  |  |  |
| **Participant** | **Frieburg Scale post:** | **Q1** | **Q2** | **Q3** | **Q4** | **Q5** | **Q6** | **Q7** | **Q8** | **Q9** | **Q10** | **Q11** | **Q12** | **Q13** | **Q14** | **Participant** | |  |  |  |  |  |  |  |  |  |  |  |  |  |  |  |  |  |
| **No** | **Name** |  |  |  |  |  |  |  |  |  |  |  |  | **REVERSE** | | **total** |  |  |  |  |  |  |  |  |  |  |  |  |  |  |  |  |  |  |
| 1 |  | 4 | 4 | 4 | 3 | 3 | 4 | 4 | 4 | 3 | 4 | 3 | 4 | 3 | 2 | 49 |  |  |  |  |  |  |  |  |  |  |  |  |  |  |  |  |  |  |
| 2 |  | 4 | 3 | 3 | 3 | 4 | 3 | 4 | 4 | 4 | 3 | 3 | 4 | 4 | 1 | 47 |  |  |  |  |  |  |  |  |  |  |  |  |  |  |  |  |  |  |
| 4 |  | 3 | 4 | 3 | 2 | 3 | 2 | 3 | 3 | 3 | 3 | 2 | 2 | 3 | 3 | 39 |  |  |  |  |  |  |  |  |  |  |  |  |  |  |  |  |  |  |
| 5 |  | 4 | 3 | 3 | 3 | 3 | 3 | 3 | 4 | 3 | 3 | 4 | 3 | 1 | 3 | 43 |  |  |  |  |  |  |  |  |  |  |  |  |  |  |  |  |  |  |
| 6 |  | 4 | 3 | 3 | 4 | 3 | 4 | 4 | 4 | 3 | 4 | 4 | 4 | 3 | 2 | 49 |  |  |  |  |  |  |  |  |  |  |  |  |  |  |  |  |  |  |
| 8 |  | 3 | 3 | 3 | 3 | 3 | 2 | 3 | 3 | 2 | 3 | 3 | 3 | 3 | 2 | 39 |  |  |  |  |  |  |  |  |  |  |  |  |  |  |  |  |  |  |
| 9 |  | 3 | 3 | 3 | 3 | 3 | 3 | 3 | 3 | 3 | 2 | 3 | 2 | 3 | 3 | 40 |  |  |  |  |  |  |  |  |  |  |  |  |  |  |  |  |  |  |
| 10 |  | 4 | 3 | 3 | 3 | 4 | 2 | 4 | 3 | 3 | 4 | 2 | 4 | 2 | 1 | 42 |  |  |  |  |  |  |  |  |  |  |  |  |  |  |  |  |  |  |
| 12 |  | 4 | 3 | 4 | 3 | 4 | 3 | 4 | 4 | 3 | 3 | 2 | 4 | 3 | 4 | 48 |  |  |  |  |  |  |  |  |  |  |  |  |  |  |  |  |  |  |
| 13 |  | 3 | 3 | 3 | 3 | 3 | 3 | 3 | 2 | 3 | 3 | 2 | 2 | 3 | 3 | 39 |  |  |  |  |  |  |  |  |  |  |  |  |  |  |  |  |  |  |
| 14 |  | 3 | 3 | 4 | 3 | 4 | 2 | 3 | 3 | 2 | 3 | 3 | 2 | 3 | 2 | 40 |  |  |  |  |  |  |  |  |  |  |  |  |  |  |  |  |  |  |
| 15 |  | 3 | 3 | 3 | 2 | 3 | 3 | 3 | 2 | 2 | 2 | 3 | 3 | 3 | 2 | 37 |  |  |  |  |  |  |  |  |  |  |  |  |  |  |  |  |  |  |
| 16 |  | 3 | 3 | 2 | 3 | 3 | 3 | 3 | 2 | 2 | 2 | 2 | 2 | 3 | 1 | 34 |  |  |  |  |  |  |  |  |  |  |  |  |  |  |  |  |  |  |
|  | **Group Mean** |  |  |  |  |  |  |  |  |  |  |  |  |  |  | **42** |  |  |  |  |  |  |  |  |  |  |  |  |  |  |  |  |  |  |
|  |  |  |  |  |  |  |  |  |  |  |  |  |  |  |  |  |  |  |  |  |  |  |  |  |  |  |  |  |  |  |  |  |  |  |
| **Participant** | **Self-compassion Scale .PRE** | **Q1/R** | **Q8/R** | **11/R** | **Q16/R** | **Q21/R** | **S-JUDGE** | **Q2/R** | **Q6/R** | **Q20/R** | **Q24/R** | **OVr-IdTFY** | **Q4/R** | **Q13/R** | **Q18/R** | **Q25/R** | **Isol** | **Q3** | **Q7** | **Q10** | **Q15** | **CMN. H** | **Q5** | **Q12** | **Q19** | **Q23** | **Q26** | **Self-K** | **Q9** | **Q14** | **Q17** | **Q22** | **MNDFNESS** | **SC TOTAL** |
| 1 |  | 1 | 5 | 1 | 1 | 2 | **2** | 1 | 1 | 1 | 1 | **1** | 1 | 1 | 2 | 1 | 2 | 5 | 3 | 3 | 4 | 3.75 | 5 | 4 | 5 | 3 | 3 | 4 | 2 | 1 | 3 | 3 | 2.25 | 2.50 |
| 2 |  | 2 | 2 | 3 | 3 | 3 | **2.6** | 4 | 4 | 4 | 3 | **3.75** | 4 | 4 | 4 | 4 | 4 | 4 | 5 | 4 | 4 | 4.25 | 4 | 1 | 2 | 3 | 3 | 2.6 | 2 | 3 | 3 | 4 | 3 | 3.37 |
| 4 |  | 1 | 1 | 1 | 1 | 1 | **1** | 1 | 1 | 1 | 1 | **1** | 3 | 1 | 3 | 1 | 2 | 4 | 3 | 2 | 2 | 2.75 | 3 | 1 | 2 | 2 | 2 | 2 | 2 | 2 | 1 | 2 | 1.75 | 1.75 |
| 5 |  | 2 | 3 | 4 | 2 | 3 | **2.8** | 3 | 4 | 4 | 2 | **3.25** | 3 | 2 | 4 | 2 | 2.75 | 5 | 5 | 3 | 4 | 4.25 | 1 | 1 | 2 | 3 | 4 | 2 | 4 | 5 | 4 | 2 | 3.75 | 3.13 |
| 6 |  | 4 | 3 | 4 | 3 | 3 | **3.4** | 4 | 3 | 4 | 4 | **3.75** | 3 | 2 | 4 | 3 | 3 | 3 | 2 | 3 | 3 | 2.75 | 3 | 2 | 2 | 3 | 3 | 2.6 | 3 | 3 | 3 | 3 | 3 | 3.08 |
| 8 |  | 2 | 2 | 1 | 2 | 3 | **2** | 2 | 1 | 2 | 4 | **2.25** | 2 | 2 | 2 | 2 | 2 | 5 | 3 | 3 | 3 | 3.5 | 3 | 2 | 3 | 3 | 3 | 2.8 | 3 | 3 | 3 | 1 | 2.5 | 2.51 |
| 9 |  | 2 | 2 | 2 | 2 | 3 | **2.2** | 2 | 4 | 3 | 4 | **3.25** | 4 | 2 | 2 | 2 | 2.5 | 4 | 5 | 3 | 3 | 3.75 | 3 | 2 | 3 | 3 | 3 | 2.8 | 3 | 3 | 3 | 3 | 3 | 2.92 |
| 10 |  | 3 | 5 | 5 | 5 | 5 | **4.6** | 4 | 5 | 5 | 4 | **4.5** | 3 | 5 | 5 | 5 | 4.5 | 5 | 3 | 5 | 3 | 4 | 4 | 3 | 4 | 4 | 5 | 4 | 4 | 4 | 4 | 5 | 4.25 | 4.31 |
| 12 |  | 1 | 2 | 2 | 2 | 3 | **2** | 1 | 2 | 3 | 4 | **2.5** | 2 | 3 | 4 | 2 | 2.75 | 5 | 3 | 2 | 3 | 3.25 | 2 | 2 | 3 | 1 | 3 | 2.2 | 3 | 5 | 4 | 4 | 4 | 2.78 |
| 13 |  | 2 | 2 | 3 | 3 | 3 | **2.6** | 2 | 3 | 2 | 2 | **2.25** | 3 | 3 | 3 | 3 | 3 | 3 | 4 | 3 | 4 | 3.5 | 4 | 3 | 3 | 3 | 3 | 3.2 | 4 | 4 | 3 | 4 | 3.75 | 3.05 |
| 14 |  | 3 | 3 | 2 | 3 | 3 | **2.8** | 3 | 2 | 4 | 4 | **3.25** | 5 | 4 | 4 | 3 | 4 | 4 | 4 | 4 | 4 | 4 | 2 | 3 | 3 | 3 | 2 | 2.6 | 5 | 5 | 4 | 3 | 4.25 | 3.48 |
| 15 |  | 1 | 1 | 1 | 3 | 2 | **1.6** | 1 | 5 | 1 | 1 | **2** | 1 | 4 | 1 | 1 | 1.75 | 4 | 1 | 2 | 1 | 2 | 1 | 1 | 1 | 1 | 1 | 1 | 5 | 3 | 1 | 1 | 2.5 | 1.81 |
| 16 |  | 3 | 2 | 2 | 2 | 3 | **2.4** | 2 | 3 | 2 | 2 | **2.25** | 2 | 3 | 3 | 2 | 2.5 | 4 | 3 | 3 | 4 | 3.5 | 3 | 3 | 3 | 3 | 3 | 3 | 2 | 2 | 3 | 3 | 2.5 | 2.69 |
|  | **Group Overall mean** |  |  |  |  |  |  |  |  |  |  | **2.89** |  |  |  |  | **2.89** |  |  |  |  |  |  |  |  |  |  | **2.69** |  |  |  |  | **3.31** | **2.88** |
| **Participant** | **Self-compassion Scale.POST** | **Q1/R** | **Q8/R** | **11/R** | **Q16/R** | **Q21/R** | **S-JUDGE** | **Q2/R** | **Q6/R** | **Q20/R** | **Q24/R** | **Over-Identify** | **Q4/R** | **Q13/R** | **Q18/R** | **Q25/R** | **Isolation** | **Q3** | **Q7** | **Q10** | **Q15** | **CMN. H** | **Q5** | **Q12** | **Q19** | **Q23** | **Q26** | **Self-K** | **Q9** | **Q14** | **Q17** | **Q22** | **MNDFNESS** | **SC TOTAL** |
| 1 |  | 3 | 4 | 4 | 1 | 5 | **3.4** | 2 | 1 | 1 | 2 | **1.5** | 2 | 1 | 1 | 1 | 1.25 | 4 | 5 | 5 | 5 | 4.75 | 5 | 5 | 5 | 4 | 5 | 4.8 | 4 | 5 | 5 | 4 | 4.5 | 3.37 |
| 2 |  | 4 | 3 | 3 | 3 | 3 | **3.2** | 4 | 4 | 3 | 3 | **3.5** | 4 | 4 | 4 | 4 | 4 | 4 | 4 | 4 | 4 | 4 | 4 | 4 | 4 | 3 | 4 | 3.8 | 4 | 3 | 4 | 4 | 3.75 | 3.71 |
| 4 |  | 3 | 3 | 3 | 3 | 4 | **3.2** | 3 | 3 | 3 | 3 | **3** | 3 | 4 | 4 | 3 | 3.5 | 4 | 4 | 4 | 4 | 4 | 4 | 4 | 4 | 3 | 4 | 3.8 | 3 | 4 | 3 | 4 | 3.5 | 3.50 |
| 5 |  | 4 | 4 | 4 | 3 | 4 | **3.8** | 5 | 3 | 4 | 5 | **4.25** | 4 | 5 | 4 | 3 | 4 | 5 | 5 | 4 | 4 | 4.5 | 3 | 3 | 3 | 4 | 4 | 3.4 | 5 | 5 | 3 | 3 | 4 | 3.99 |
| 6 |  | 4 | 4 | 5 | 4 | 4 | **4.2** | 4 | 4 | 4 | 4 | **4** | 4 | 4 | 4 | 4 | 4 | 5 | 5 | 5 | 5 | 5 | 4 | 4 | 4 | 5 | 4 | 4.2 | 5 | 5 | 5 | 4 | 4.75 | 4.36 |
| 8 |  | 2 | 2 | 3 | 3 | 2 | **2.4** | 2 | 3 | 3 | 4 | **3** | 3 | 2 | 3 | 4 | 3 | 5 | 4 | 4 | 3 | 4 | 4 | 2 | 3 | 3 | 4 | 3.2 | 4 | 3 | 5 | 3 | 3.75 | 3.23 |
| 9 |  | 3 | 3 | 3 | 3 | 3 | **3** | 4 | 3 | 3 | 4 | **3.5** | 3 | 2 | 3 | 3 | 2.75 | 4 | 4 | 4 | 4 | 4 | 4 | 3 | 4 | 4 | 4 | 3.8 | 4 | 4 | 3 | 3 | 3.5 | 3.43 |
| 10 |  | 3 | 4 | 4 | 5 | 4 | **4** | 5 | 3 | 4 | 4 | **4** | 3 | 4 | 5 | 4 | 4 | 5 | 4 | 4 | 4 | 4.25 | 2 | 2 | 2 | 5 | 4 | 3 | 4 | 5 | 5 | 4 | 4.5 | 3.96 |
| 12 |  | 2 | 2 | 3 | 3 | 4 | **2.8** | 4 | 1 | 2 | 4 | **2.75** | 3 | 4 | 4 | 3 | 3.5 | 5 | 4 | 4 | 4 | 4.25 | 4 | 3 | 3 | 3 | 3 | 3.2 | 4 | 4 | 3 | 4 | 3.75 | 3.38 |
| 13 |  | 4 | 4 | 3 | 4 | 3 | **3.6** | 3 | 4 | 4 | 4 | **3.75** | 4 | 4 | 4 | 4 | 4 | 4 | 5 | 4 | 4 | 4.25 | 5 | 4 | 4 | 4 | 3 | 4 | 3 | 4 | 4 | 4 | 3.75 | 3.89 |
| 14 |  | 3 | 2 | 3 | 3 | 2 | **2.6** | 4 | 3 | 4 | 4 | **3.75** | 4 | 4 | 4 | 3 | 3.75 | 4 | 4 | 4 | 3 | 3.75 | 3 | 2 | 3 | 3 | 2 | 2.6 | 5 | 4 | 4 | 3 | 4 | 3.41 |
| 15 |  | 3 | 2 | 2 | 3 | 3 | **2.6** | 2 | 3 | 3 | 4 | **3** | 3 | 2 | 4 | 3 | 3 | 3 | 4 | 4 | 4 | 3.75 | 4 | 4 | 4 | 3 | 3 | 3.6 | 3 | 4 | 4 | 3 | 3.5 | 3.24 |
| 16 |  | 2 | 3 | 3 | 3 | 3 | **2.8** | 3 | 3 | 3 | 4 | **3.25** | 3 | 2 | 3 | 3 | 2.75 | 3 | 3 | 3 | 3 | 3 | 3 | 3 | 3 | 3 | 3 | 3 | 3 | 3 | 3 | 3 | 3 | 2.97 |
|  | **Group Overall mean** |  |  |  |  |  |  |  |  |  |  | 3.326923077 |  |  |  |  | 3.346153846 |  |  |  |  |  |  |  |  |  |  | 3.569230769 |  |  |  |  | 3.865384615 | **3.57** |
