# Supplementary material for: Caring for the caregivers: Evaluation of the effect of an eight-week pilot mindful self-compassion (MSC) training program on nurses’ compassion fatigue and resilience
Source: PLoS One. 2018 Nov 21;13(11):e0207261. doi: 10.1371/journal.pone.0207261 (PMC6248952; doi:10.1371/journal.pone.0207261)
Supplement: S2 Table — (DOCX) [file pone.0207261.s002.docx]

| **Quantitative Data Scores** |  |  |  |  |  |  |  |  |  |  |  |  |  |  |  |  |  |  |  |  |  |  |  |  |  |  |  |  |  |  |  |  |  |  |
| --- | --- | --- | --- | --- | --- | --- | --- | --- | --- | --- | --- | --- | --- | --- | --- | --- | --- | --- | --- | --- | --- | --- | --- | --- | --- | --- | --- | --- | --- | --- | --- | --- | --- | --- |
| **Partcpt.** | **PROQOL PRE** | **Q3** | **Q6** | **Q12** | **Q16** | **Q18** | **Q20** | **Q22** | **Q24** | **Q27** | **Q30** | **Compassion satisfaction** | **Q1 R.** | **Q4 R** | **Q15 R** | **Q17.R** | **Q29.R** | **Q8** | **Q10** | **Q19** | **Q21** | **Q26** | **Burnout** | **Q2** | **Q5** | **Q7** | **Q9** | **Q11** | **Q13** | **Q14** | **Q23** | **Q25** | **Q28** | **2nd.Traumatic Stress** |
| 1 |  | 4 | 4 | 4 | 4 | 4 | 4 | 4 | 3 | 1 | 4 | **36** | 1 | 2 | 3 | 4 | 1 | 1 | 5 | 5 | 4 | 4 | **30** | 5 | 4 | 4 | 2 | 2 | 3 | 2 | 1 | 1 | 1 | **25** |
| 2 |  | 5 | 3 | 4 | 3 | 3 | 4 | 4 | 4 | 2 | 3 | **35** | 2 | 1 | 2 | 3 | 3 | 2 | 4 | 3 | 3 | 5 | **28** | 2 | 2 | 2 | 3 | 3 | 2 | 2 | 2 | 2 | 1 | **21** |
| 4 |  | 5 | 4 | 5 | 4 | 3 | 4 | 3 | 4 | 2 | 4 | **38** | 2 | 2 | 3 | 3 | 2 | 3 | 4 | 4 | 4 | 5 | **32** | 4 | 3 | 4 | 3 | 3 | 3 | 3 | 2 | 3 | 4 | **32** |
| 5 |  | 5 | 4 | 5 | 4 | 3 | 4 | 3 | 3 | 2 | 4 | **37** | 1 | 2 | 1 | 3 | 2 | 2 | 2 | 3 | 3 | 3 | **22** | 5 | 4 | 2 | 2 | 2 | 2 | 2 | 1 | 2 | 3 | **25** |
| 6 |  | 4 | 4 | 4 | 4 | 3 | 3 | 3 | 4 | 4 | 4 | **37** | 2 | 4 | 2 | 2 | 2 | 2 | 3 | 4 | 5 | 5 | **31** | 4 | 2 | 2 | 2 | 4 | 2 | 2 | 2 | 2 | 1 | **23** |
| 8 |  | 5 | 4 | 4 | 4 | 3 | 3 | 3 | 4 | 3 | 4 | **37** | 2 | 3 | 2 | 3 | 1 | 2 | 3 | 4 | 3 | 4 | **27** | 4 | 3 | 4 | 3 | 4 | 3 | 3 | 4 | 2 | 2 | **32** |
| 9 |  | 5 | 3 | 5 | 3 | 4 | 3 | 4 | 4 | 4 | 4 | **39** | 2 | 2 | 3 | 3 | 2 | 3 | 2 | 5 | 5 | 4 | **31** | 4 | 3 | 4 | 3 | 3 | 1 | 2 | 1 | 1 | 2 | **24** |
| 10 |  | 4 | 4 | 4 | 3 | 5 | 4 | 4 | 4 | 4 | 4 | **40** | 1 | 1 | 1 | 2 | 2 | 2 | 2 | 4 | 4 | 5 | **24** | 5 | 2 | 4 | 3 | 4 | 1 | 2 | 3 | 1 | 3 | **28** |
| 12 |  | 5 | 3 | 4 | 3 | 4 | 4 | 3 | 3 | 2 | 5 | **36** | 2 | 2 | 2 | 3 | 2 | 4 | 3 | 3 | 4 | 4 | **29** | 5 | 4 | 5 | 3 | 3 | 2 | 2 | 2 | 2 | 5 | **33** |
| 13 |  | 5 | 3 | 4 | 3 | 3 | 4 | 5 | 4 | 4 | 4 | **39** | 2 | 2 | 2 | 3 | 1 | 4 | 3 | 5 | 3 | 4 | **29** | 4 | 4 | 3 | 3 | 3 | 3 | 3 | 2 | 2 | 3 | **30** |
| 14 |  | 4 | 4 | 4 | 3 | 3 | 2 | 4 | 4 | 2 | 4 | **34** | 1 | 2 | 2 | 3 | 3 | 2 | 3 | 3 | 3 | 4 | **26** | 2 | 4 | 2 | 2 | 3 | 1 | 1 | 2 | 2 | 3 | **22** |
| 15 |  | 5 | 3 | 5 | 2 | 3 | 4 | 4 | 5 | 2 | 4 | **37** | 4 | 3 | 4 | 5 | 3 | 2 | 4 | 5 | 5 | 5 | **40** | 2 | 5 | 1 | 3 | 4 | 3 | 3 | 3 | 2 | 3 | **29** |
| 16 |  | 5 | 5 | 5 | 4 | 5 | 5 | 5 | 5 | 4 | 5 | **48** | 2 | 2 | 2 | 3 | 1 | 2 | 3 | 4 | 5 | 5 | **29** | 5 | 4 | 3 | 3 | 3 | 3 | 3 | 3 | 2 | 1 | **30** |
|  |  |  |  |  |  |  |  |  |  |  |  | **37.92** |  |  |  |  |  |  |  |  |  |  | **29.08** |  |  |  |  |  |  |  |  |  |  | **27.23** |
|  |  |  |  |  |  |  |  |  |  |  |  |  |  |  |  |  |  |  |  |  |  |  |  |  |  |  |  |  |  |  |  |  |  |  |
|  |  |  |  |  |  |  |  |  |  |  |  |  |  |  |  |  |  |  |  |  |  |  |  |  |  |  |  |  |  |  |  |  |  |  |
|  |  |  |  |  |  |  |  |  |  |  |  |  |  |  |  |  |  |  |  |  |  |  |  |  |  |  |  |  |  |  |  |  |  |  |
| **Partcpt.** | **PROQOL Post** | **Q3** | **Q6** | **Q12** | **Q16** | **Q18** | **Q20** | **Q22** | **Q24** | **Q27** | **Q30** | **Compassion satisfaction** | **Q1 R.** | **Q4 R** | **Q15 R** | **Q17.R** | **Q29.R** | **Q8** | **Q10** | **Q19** | **Q21** | **Q26** | **Burnout** | **Q2** | **Q5** | **Q7** | **Q9** | **Q11** | **Q13** | **Q14** | **Q23** | **Q25** | **Q28** | **2Nd. Traumatic Stress** |
| 1 |  | 5 | 5 | 5 | 5 | 5 | 3 | 5 | 5 | 1 | 5 | **44** | 1 | 1 | 1 | 3 | 1 | 1 | 3 | 4 | 3 | 1 | **19** | 5 | 3 | 2 | 3 | 1 | 1 | 3 | 1 | 1 | 1 | **21** |
| 2 |  | 5 | 3 | 4 | 3 | 3 | 4 | 3 | 3 | 3 | 3 | **34** | 2 | 1 | 3 | 3 | 3 | 2 | 2 | 3 | 3 | 4 | **26** | 2 | 2 | 2 | 2 | 3 | 2 | 2 | 2 | 1 | 2 | **20** |
| 4 |  | 4 | 4 | 4 | 4 | 3 | 4 | 4 | 4 | 3 | 4 | **38** | 2 | 2 | 2 | 3 | 2 | 2 | 2 | 3 | 3 | 4 | **25** | 3 | 3 | 3 | 2 | 4 | 2 | 3 | 2 | 2 | 2 | **26** |
|  |  | 5 | 4 | 5 | 4 | 3 | 3 | 4 | 4 | 3 | 5 | **40** | 2 | 2 | 1 | 3 | 1 | 1 | 1 | 3 | 3 | 2 | **19** | 4 | 3 | 1 | 2 | 2 | 2 | 2 | 1 | 1 | 2 | **20** |
| 6 |  | 5 | 5 | 5 | 5 | 4 | 3 | 4 | 5 | 3 | 5 | **44** | 1 | 1 | 1 | 1 | 1 | 2 | 3 | 3 | 3 | 3 | **19** | 5 | 2 | 2 | 2 | 3 | 2 | 1 | 1 | 1 | 1 | **20** |
| 8 |  | 5 | 4 | 4 | 4 | 3 | 4 | 4 | 4 | 3 | 4 | **39** | 2 | 1 | 2 | 3 | 1 | 1 | 3 | 3 | 3 | 5 | **24** | 5 | 3 | 3 | 3 | 3 | 2 | 2 | 2 | 2 | 3 | **28** |
| 9 |  | 5 | 4 | 5 | 4 | 4 | 4 | 4 | 4 | 4 | 5 | **43** | 2 | 2 | 2 | 2 | 2 | 1 | 1 | 3 | 3 | 3 | **21** | 3 | 2 | 2 | 2 | 4 | 1 | 1 | 1 | 1 | 2 | **19** |
| 10 |  | 5 | 4 | 4 | 3 | 4 | 4 | 4 | 4 | 4 | 4 | **40** | 2 | 3 | 1 | 2 | 2 | 2 | 1 | 3 | 3 | 4 | **23** | 2 | 3 | 2 | 3 | 2 | 2 | 2 | 1 | 1 | 1 | **19** |
| 12 |  | 5 | 4 | 4 | 4 | 4 | 5 | 4 | 3 | 3 | 5 | **41** | 1 | 1 | 1 | 2 | 2 | 2 | 2 | 3 | 4 | 3 | **21** | 5 | 3 | 4 | 2 | 4 | 2 | 2 | 2 | 2 | 2 | **28** |
| 13 |  | 5 | 3 | 5 | 4 | 4 | 4 | 5 | 5 | 4 | 4 | **43** | 2 | 1 | 2 | 2 | 1 | 2 | 2 | 3 | 3 | 3 | **21** | 4 | 4 | 3 | 2 | 3 | 3 | 3 | 2 | 1 | 2 | **27** |
| 14 |  | 4 | 4 | 4 | 3 | 4 | 2 | 4 | 5 | 2 | 4 | **36** | 2 | 2 | 3 | 3 | 2 | 2 | 3 | 3 | 3 | 4 | **27** | 3 | 4 | 2 | 3 | 3 | 2 | 2 | 1 | 1 | 2 | **23** |
| 15 |  | 5 | 5 | 5 | 4 | 5 | 4 | 5 | 5 | 4 | 5 | **47** | 2 | 2 | 2 | 3 | 1 | 3 | 3 | 3 | 5 | 5 | **29** | 5 | 3 | 3 | 4 | 2 | 3 | 3 | 2 | 2 | 3 | **30** |
| 16 |  | 5 | 5 | 5 | 4 | 4 | 5 | 5 | 5 | 4 | 5 | **47** | 2 | 2 | 2 | 2 | 1 | 2 | 2 | 4 | 4 | 5 | **26** | 4 | 3 | 3 | 3 | 3 | 3 | 4 | 2 | 2 | 2 | **29** |
|  |  |  |  |  |  |  |  |  |  |  |  | **41.23** |  |  |  |  |  |  |  |  |  |  | **23.08** |  |  |  |  |  |  |  |  |  |  | **23.85** |
