# Supplementary material for: Caring for the caregivers: Evaluation of the effect of an eight-week pilot mindful self-compassion (MSC) training program on nurses’ compassion fatigue and resilience
Source: PLoS One. 2018 Nov 21;13(11):e0207261. doi: 10.1371/journal.pone.0207261 (PMC6248952; doi:10.1371/journal.pone.0207261)
Supplement: S3 Table — (DOCX) [file pone.0207261.s003.docx]

| **Quantitative Data Scores** |  |  |  |  |  |  |  |  |  |  |  |  |  |  |  |  |  |  |  |  |  |  |  |  |  |  |  |
| --- | --- | --- | --- | --- | --- | --- | --- | --- | --- | --- | --- | --- | --- | --- | --- | --- | --- | --- | --- | --- | --- | --- | --- | --- | --- | --- | --- |
| **Partcpt** | **.Resilience.PRE.** | **Q1** | **Q2** | **Q3** | **Q4** | **Q5** | **Q6** | **Q7** | **Q8** | **Q9** | **Q10** | **Q11** | **Q12** | **Q13** | **Q14** | **Q15** | **Q16** | **Q17** | **Q18** | **Q19** | **Q20** | **Q21** | **Q22** | **Q23** | **Q24** | **Q25** | **total score** |
| 1 |  | 4 | 4 | 4 | 4 | 4 | 4 | 3 | 2 | 1 | 4 | 4 | 4 | 3 | 3 | 4 | 3 | 3 | 3 | 2 | 4 | 2 | 2 | 2 | 2 | 4 | 79 |
| 2 |  | 4 | 4 | 3 | 2 | 3 | 2 | 2 | 4 | 3 | 3 | 3 | 4 | 3 | 2 | 3 | 3 | 2 | 2 | 2 | 2 | 2 | 3 | 2 | 2 | 3 | 68 |
| 4 |  | 4 | 4 | 2 | 2 | 3 | 3 | 2 | 2 | 2 | 4 | 2 | 3 | 3 | 2 | 1 | 2 | 3 | 1 | 2 | 2 | 3 | 3 | 2 | 3 | 2 | 62 |
| 5 |  | 3 | 4 | 4 | 3 | 3 | 3 | 4 | 4 | 4 | 3 | 2 | 1 | 2 | 2 | 3 | 2 | 3 | 3 | 4 | 3 | 3 | 2 | 2 | 1 | 2 | 62 |
| 6 |  | 2 | 3 | 3 | 2 | 2 | 2 | 2 | 2 | 3 | 3 | 2 | 2 | 2 | 3 | 2 | 2 | 2 | 2 | 2 | 2 | 2 | 2 | 2 | 2 | 3 | 56 |
| 8 |  | 2 | 2 | 4 | 3 | 3 | 3 | 3 | 3 | 2 | 4 | 4 | 4 | 3 | 3 | 3 | 2 | 2 | 2 | 3 | 3 | 3 | 3 | 2 | 4 | 4 | 74 |
| 9 |  | 3 | 3 | 2 | 3 | 2 | 2 | 2 | 3 | 3 | 4 | 3 | 3 | 3 | 2 | 3 | 2 | 3 | 2 | 2 | 3 | 3 | 3 | 2 | 3 | 3 | 67 |
| 10 |  | 4 | 4 | 2 | 4 | 3 | 3 | 3 | 4 | 3 | 3 | 4 | 3 | 3 | 4 | 3 | 3 | 4 | 1 | 3 | 2 | 4 | 4 | 4 | 3 | 4 | 82 |
| 12 |  | 4 | 3 | 4 | 4 | 2 | 4 | 3 | 4 | 4 | 4 | 4 | 3 | 3 | 2 | 2 | 2 | 2 | 1 | 1 | 3 | 2 | 1 | 1 | 3 | 2 | 68 |
| 13 |  | 2 | 4 | 3 | 2 | 3 | 3 | 2 | 3 | 3 | 4 | 3 | 2 | 4 | 4 | 3 | 2 | 3 | 2 | 2 | 2 | 2 | 2 | 2 | 3 | 3 | 68 |
| 14 |  | 4 | 4 | 3 | 3 | 3 | 3 | 3 | 4 | 4 | 3 | 3 | 3 | 3 | 3 | 3 | 2 | 3 | 2 | 3 | 3 | 3 | 3 | 3 | 3 | 4 | 78 |
| 15 |  | 1 | 0 | 2 | 3 | 4 | 4 | 4 | 4 | 4 | 3 | 2 | 0 | 2 | 2 | 2 | 1 | 1 | 3 | 0 | 3 | 0 | 1 | 2 | 2 | 3 | 53 |
| 16 |  | 3 | 3 | 2 | 2 | 2 | 2 | 2 | 3 | 4 | 3 | 3 | 3 | 3 | 2 | 3 | 2 | 2 | 2 | 2 | 2 | 3 | 2 | 2 | 2 | 3 | 62 |
|  | **Group Mean Score** |  |  |  |  |  |  |  |  |  |  |  |  |  |  |  |  |  |  |  |  |  |  |  |  |  | **67.62** |
| **Partcpt** | **.Resilience.POST** | **Q1** | **Q2** | **Q3** | **Q4** | **Q5** | **Q6** | **Q7** | **Q8** | **Q9** | **Q10** | **Q11** | **Q12** | **Q13** | **Q14** | **Q15** | **Q16** | **Q17** | **Q18** | **Q19** | **Q20** | **Q21** | **Q22** | **Q23** | **Q24** | **Q25** | **total score** |
| 1 |  | 4 | 4 | 4 | 4 | 4 | 4 | 4 | 3 | 2 | 4 | 4 | 4 | 4 | 4 | 4 | 2 | 4 | 4 | 3 | 4 | 3 | 3 | 3 | 4 | 4 | 91 |
| 2 |  | 3 | 4 | 4 | 4 | 4 | 3 | 4 | 4 | 3 | 3 | 4 | 4 | 4 | 3 | 3 | 3 | 3 | 3 | 3 | 2 | 3 | 3 | 2 | 3 | 3 | 82 |
| 4 |  | 3 | 4 | 2 | 3 | 3 | 2 | 3 | 2 | 3 | 4 | 3 | 3 | 4 | 3 | 2 | 3 | 3 | 2 | 3 | 2 | 2 | 3 | 3 | 3 | 3 | 71 |
| 5 |  | 4 | 4 | 4 | 3 | 2 | 3 | 2 | 4 | 4 | 4 | 3 | 4 | 4 | 3 | 3 | 2 | 4 | 2 | 4 | 3 | 3 | 3 | 2 | 1 | 2 | 77 |
| 6 |  | 3 | 4 | 4 | 3 | 4 | 3 | 3 | 4 | 4 | 4 | 4 | 4 | 4 | 4 | 3 | 3 | 3 | 3 | 3 | 3 | 3 | 3 | 4 | 3 | 4 | 87 |
| 8 |  | 2 | 3 | 4 | 3 | 3 | 3 | 3 | 3 | 4 | 4 | 3 | 4 | 3 | 2 | 2 | 2 | 2 | 2 | 3 | 3 | 3 | 2 | 3 | 3 | 4 | 73 |
| 9 |  | 3 | 4 | 3 | 4 | 3 | 3 | 3 | 3 | 4 | 4 | 3 | 4 | 4 | 4 | 4 | 4 | 4 | 3 | 3 | 3 | 4 | 3 | 3 | 3 | 4 | 87 |
| 10 |  | 4 | 4 | 3 | 4 | 4 | 3 | 3 | 4 | 4 | 3 | 3 | 4 | 4 | 4 | 3 | 3 | 4 | 2 | 4 | 4 | 3 | 3 | 3 | 4 | 3 | 87 |
| 12 |  | 4 | 4 | 4 | 4 | 4 | 4 | 4 | 4 | 4 | 4 | 4 | 4 | 4 | 3 | 3 | 3 | 4 | 2 | 2 | 4 | 4 | 3 | 2 | 4 | 4 | 90 |
| 13 |  | 3 | 4 | 3 | 3 | 3 | 3 | 2 | 3 | 3 | 4 | 3 | 4 | 4 | 3 | 3 | 2 | 3 | 2 | 3 | 3 | 4 | 3 | 3 | 4 | 4 | 79 |
| 14 |  | 3 | 4 | 3 | 3 | 3 | 2 | 2 | 4 | 4 | 4 | 3 | 2 | 3 | 3 | 3 | 3 | 3 | 1 | 3 | 3 | 2 | 3 | 3 | 3 | 4 | 74 |
| 15 |  | 3 | 3 | 2 | 3 | 3 | 3 | 2 | 3 | 3 | 3 | 2 | 3 | 3 | 2 | 2 | 2 | 3 | 2 | 2 | 3 | 2 | 2 | 3 | 2 | 4 | 65 |
| 16 |  | 3 | 4 | 2 | 3 | 3 | 3 | 3 | 4 | 4 | 4 | 4 | 4 | 4 | 3 | 4 | 4 | 4 | 1 | 2 | 3 | 3 | 3 | 3 | 3 | 3 | 81 |
|  | **Group Mean Score** |  |  |  |  |  |  |  |  |  |  |  |  |  |  |  |  |  |  |  |  |  |  |  |  |  | **80.31** |
