# Supplementary material for: Caring for the caregivers: Evaluation of the effect of an eight-week pilot mindful self-compassion (MSC) training program on nurses’ compassion fatigue and resilience
Source: PLoS One. 2018 Nov 21;13(11):e0207261. doi: 10.1371/journal.pone.0207261 (PMC6248952; doi:10.1371/journal.pone.0207261)
Supplement: S4 Table — (DOCX) [file pone.0207261.s004.docx]

|  |  |  |  |  |  |  |  | **Wk 8 Focus group session** |
| --- | --- | --- | --- | --- | --- | --- | --- | --- |
|  |  |  |  |  |  |  |  | ***what was your experience of taking this MSC training course?*** |
| **P.I.D.** | **Positive Mental States (B)** | **Reduced Stress.(C)** | **Enhanced Coping(D)** | **Acceptance. (E)** | **Mindful Awareness (F)** | **Difficulty practicing (G)** | **Less Self-critical (H)** | **RESPONSE** |
| **1** | Happier (B3) More Content (B8) More relaxed (B9) sleeping better (B7) | less Stressed =(C1) | Better able to cope with working day.=(D1) | more Acceptance ofExterior circumstances(E1)More Acceotance of Interior Affect(E2) |  |  | **Less Self-critical (H 1)** | feeling happier(B3), more content (B8)with myself (H1),less stressed(C1),or uptight,learning to breathe through difficult situations &emotions(E1),(E2),sleeping better (B7), more relaxed,better(B9) able to cope with working days demands (D1). |
| **2** | More peaceful (B1) More Relaxed(B9) Improved Concentration(B2) | less Stress =(C1) | Better Coping in work.=(D1) better Coping Personal Life(D2) | More Acceotance of Interior Affect(E2) | Less distracted (F2) |  | **Less Self-critical (H 1)** | Letting more go, not fearful of opening up(E2), acknowledging more and finding it helpful, accepting things and lettings go (E2),feeling more peaceful (B1) and relaxed(B9),not getting worked up or stressed,as much(C1), better concentration (B2),can see how distracted I was(F2), Not giving 100% anyplace & not feeling bad about it,(H1) more satisified with myself in work & outside,(H1) |
| **3** |  | less Stress =(C1) | Better Coping in work.=(D1 Better )Coping Personal Life(D2) | more Acceptance of Exterior circumstances(E1) | More Mindful & aware (F1) |  | **Less Self-critical (H 1)** | I find the informal practice helpful day to day in work, less stress.(C1) Also when out of work walking etc(D1)(D2). I enjoyed the course & Intend to continue to practice Both formal & Informal,i have more mindful of accepting negative situations,(E1)(F1) I found affectionate breathing very benefical, it helped me to be less selfcritical & give myself a break(H1) |
| **4** | more calm=(B6) more confident (B10) |  | Better Coping in work.=(D1) Better Coping Personal Life(D2) |  |  |  |  | better able to cope with difficulties(D1)(D2), better posture,more confident at work (B10),feeling calmer(B6), Better atmosphere at home MSC, has brought a positive vibe to my life.& relationshios , feeling calmer,(D2) |
| **5** |  |  | Better Coping in work.=(D1) | more Acceptance ofExterior circumstances(E1) |  |  | **Less Self-critical (H 1)** | using informal practice at work allows me to step back from a situation,accepting it for what it(E1) is,Ifelt writing the compassionate letter to myself difficult because I do feel at times shame and not good enough. Putting it on paper and reading it over. It helped me to see things differently.I found the compassionate side of writting the letter easier than I thoughtit would be(H1). I now realise i just need to be more self-compassionate & less self-critical(H1) |
| **6** |  |  |  |  |  | **Falling Asleep(G1)** |  | difficult to do Self-compassion find myself falling asleep(G1) |
| **7** | more calm=(B6) |  | Better Coping in work.=(D1) | more Acceptance ofExterior circumstances(E1) | More mindful & Aware (F1) |  | **Less Self-critical (H 1)** | Becoming more aware and mindful (F1), feeling calmer,(B6)better able to cope with day to day hassle at work,(D1) more accepting of difficulties,saying to myself this too will pass(H1) |
|  | **KEY** |  |  |  |  |  |  |  |
|  | **Positive Mental States (B)** | **CODES** | **Codes** | **Codes** | **Codes** | **Codes** | **Codes** |  |
|  | More peaceful= (B1) | **Reduced Stress(C)** | **Enhanced coping (D)** | **Acceptance (E)** | **Awareness (F)** | **Difficulty practicing(G)** | **Less Self-critical (H)** |  |
|  | improved concentration=(B2) | less Stress =(C1) | Better Coping in work.=(D1) | more Acceptance ofExterior circumstances(E1) | More Mindful & aware (F1) | **Falling Asleep(G1)** | **Less Self-critical (H 1)** |  |
|  | happy =(B3) | Less exhaustion(C2) | Better Coping Personal Life(D2) | More Acceotance of Interior Affect(E2) | less Distracted(F2) | **Resistance to practice (G2)** |  |  |
|  | comforting=(B4) |  |  |  |  |  |  |  |
|  | more calm=(B6) |  |  |  |  |  |  |  |
|  | improved sleep or rest=(B7) |  |  |  |  |  |  |  |
|  | more contentment (B8) |  |  |  |  |  |  |  |
|  | more Relaxed (B9) |  |  |  |  |  |  |  |
|  | more confident (B10) |  |  |  |  |  |  |  |
|  | **P.I.D=Participant I.D.** |  |  |  |  |  |  |  |
