# Supplementary material for: Caring for the caregivers: Evaluation of the effect of an eight-week pilot mindful self-compassion (MSC) training program on nurses’ compassion fatigue and resilience
Source: PLoS One. 2018 Nov 21;13(11):e0207261. doi: 10.1371/journal.pone.0207261 (PMC6248952; doi:10.1371/journal.pone.0207261)
Supplement: S5 Table — (DOCX) [file pone.0207261.s005.docx]

|  |  |  |  |  |  |  |  | **Wk 8 Focus group session** |
| --- | --- | --- | --- | --- | --- | --- | --- | --- |
|  |  |  |  |  |  |  |  | ***what was your experience of taking this MSC training course?*** |
|  |  |  |  |  |  |  |  | **RESPONSE** |
| **P.I.D.** | **Positive Mental states (B)** | **Reduced Stress(C)** | **Enhanced Coping .** | **Acceptance(E)** | **awareness(F)** | **Difficulty Practicing(G)** | **Less Self-Critical** |  |
| **8** |  | Less exhaustion =(C2) | Better coping with work difficulty=(D1) |  |  |  |  | used practices during work , did giving and receiving compassion, while counselling clients who are going through the loss of a child,I was less exhausted(C2) after this session than I woud normally be(D1) |
| **9** |  |  |  | opening up was ok , I knew healing was happening(E2) |  | kept falling asleep (G1) |  | At first kept falling asleep during the practice(G1). But then later I,cried for a week, but I new opening up was ok that healing was happening(E2) |
| **10** | improved concentration,(B2) greater calm,=(B6) more peace=(B1), more rested=(B7) |  | more aware of situations can take a step back=(D2) |  | mind not wandering so much. More aware of situations(F1) |  |  | Mind not wandering(F2) so much concentrating (B2)better, greater calm(B6) & peace,(B1) sleeping better, more rested,(B7) more aware of situations (F1) and can take a step back |
| 11 |  |  |  |  |  | Lots of resistance.(G2) |  | Felt it difficult to concentrate on the breath, thought it would be easier & that I would be more in tune with mindfulness,feeling a lot of resistance, felt old habits returning ie over reacting (G2) |
| **12** |  | less stress =(C1) | Informal practice helpful @ work better able to deal with difficult situations=(D1) | better able to assess & accept situations(E1) | informal practice allowed me to be more mindful(F1) | Falling asleep(G1) |  | I found the infromal practice at work helpful in stressful situations (C1)it allowed me to pause take 3 breaths be mindful & assess & accept the situation,(E1) I am then better able to deal with it from there(D1), With the formal practice I find myself fallingg asleep (G1) |
| **13** | more peaceful=(B1), comforting=B4), happy=(B3) |  |  | More acknowledging and letting be (E2) | more aware of keeping myself closed, protecting myself(F2) |  | **Less Self-Critical (H 1)** | Felt more peaceful. (B1)Comforting(B4), happy (B3), I realise how much I kept closed, protecting myself , now focusing more and letting it be,(E2) (H1)acknowledging more , & more aware(F2) |
|  |  |  |  |  |  |  |  |  |
|  | **Codes** | **CODES** | **Codes** | **Codes** | **Codes** | **Codes** | **Codes** |  |
|  | **Positive Mental States (B)** | **Reduced Stress(C)** | **Enhanced coping (D)** | **Acceptance (E)** | **Awareness (F)** | **Difficulty practicing(G)** | **Less Self-Critical (H)** |  |
|  | More peaceful= (B1) | less Stress =(C1) | Coping with work difficulties= (D1) | Exterior circumstances(E1) | More mindful Aware(F1) | **Falling Asleep(G1)** | **Less Self-Critical (H 1)** |  |
|  | improved concentration=(B2) | Less exhaustion(C2) | Coping Personal Life(D2) | Interior Affect(E2) | Less distracted (F2) | **Resistance to practice (G2)** |  |  |
|  | happy =(B3) |  |  |  |  |  |  |  |
|  | comforting=(B4) |  |  |  |  |  |  |  |
|  | more calm=(B6) |  |  |  |  |  |  |  |
|  | improved sleep or rest=(B7) |  |  |  |  |  |  |  |
